# Magnetic-Driven Hydrogel Microrobots Selectively Enhance Synthetic Lethality in MTAP-Deleted Osteosarcoma

**DOI:** 10.3389/fbioe.2022.911455

**Published:** 2022-07-06

**Authors:** Haoran Mu, Chenlu Liu, Qi Zhang, Huanliang Meng, Shimin Yu, Ke Zeng, Jing Han, Xinmeng Jin, Shi Shi, Peiyao Yu, Tianlong Li, Jing Xu, Yingqi Hua

**Affiliations:** ^1^ Department of Orthopedics, Shanghai General Hospital, Shanghai Jiao Tong University School of Medicine, Shanghai, China; ^2^ Shanghai Bone Tumor Institution, Shanghai, China; ^3^ State Key Laboratory of Robotics and System, Harbin Institute of Technology, Harbin, China; ^4^ Department of Biochemistry and Molecular Cell Biology, Shanghai Key Laboratory for Tumor Microenvironment and Inflammation, Shanghai Jiao Tong University School of Medicine, Shanghai, China; ^5^ School of Basic Medical Sciences, Harbin Medical University, Harbin, China

**Keywords:** microrobots, osteosarcoma, drug delivery system, MTAP deletion, PRMT5

## Abstract

**Background:** Drugs based on synthetic lethality have advantages such as inhibiting tumor growth and affecting normal tissue *in vivo*. However, specific targets for osteosarcoma have not been acknowledged yet. In this study, a non-targeted but controllable drug delivery system has been applied to selectively enhance synthetic lethality in osteosarcoma *in vitro*, using the magnetic-driven hydrogel microrobots.

**Methods:** In this study, EPZ015666, a PRMT5 inhibitor, was selected as the synthetic lethality drug. Then, the drug was carried by hydrogel microrobots containing Fe_3_O_4_. Morphological characteristics of the microrobots were detected using electron microscopy. *In vitro* drug effect was detected by the CCK-8 assay kit, Western blotting, etc. Swimming of microrobots was observed by a timing microscope. Selective inhibition was verified by cultured tumors in an increasing magnetic field.

**Results:** Genomic mutation of MTAP deletion occurred commonly in pan-cancer in the TCGA database (nearly 10.00%) and in osteosarcoma in the TARGET database (23.86%). HOS and its derivatives, 143B and HOS/MNNG, were detected by MTAP deletion according to the CCLE database and RT-PCR. EPZ015666, the PRMT5 inhibitor, could reduce the SDMA modification and inhibition of tumor growth of 143B and HOS/MNNG. The hydrogel microrobot drug delivery system was synthesized, and the drug was stained by rhodamine. The microrobots were powered actively by a magnetic field. A simulation of the selected inhibition of microrobots was performed and lower cell viability of tumor cells was detected by adding a high dose of microrobots.

**Conclusion:** Our magnetic-driven drug delivery system could carry synthetic lethality drugs. Meanwhile, the selective inhibition of this system could be easily controlled by programming the strength of the magnetic field.

## Introduction

Some known genetic drivers found by mutational analysis have not been directly targeted, yet due to their specific molecular structure, such as undruggable oncogenes or genomic functional loss of some tumor suppressor genes. Direct drug-targeted therapy methods might be restricted in this case, while the indirect way, termed the concept of synthetic lethality, is getting attention (Huang et al., 2020). Based on functional genomic screening, synthetic lethality shows the ability to inhibit tumor growth, by targeting the side road while bypassing the side road. One of the well-known synthetic lethality pairs is PRMT5/MAT2A inhibition of MTAP-deleted tumors, mainly the homogenous deletion of MTAP (Mavrakis et al., 2016). MTAP localizes at chromosome 9p21.3 near CDKN2A, which is usually co-deleted with CDKN2A in some tumors, as reported by previous studies. MTAP deletion results in disrupted methionine metabolism, and MTAP-deleted tumors are dependent on the protein arginine methyltransferase PRMT5 (Kryukov et al., 2016; Mavrakis et al., 2016). Further, the inhibition of PRMT5 function leads to mRNA splicing deregulation and induces DNA damage (Kalev et al., 2021). The MAT2A-PRMT5 axis has also been found to create this vulnerability (Marjon et al., 2016). MTAP deletion now has been widely found in different tumors, especially in osteosarcoma (García-Castellano et al., 2002; Miyazaki et al., 2007). However, synthetic lethality shows weakness when normal cells are influenced by synthetic lethality drugs and cause strong side effects. As a result, the patient might not sustain such side effects during or after treatment. By comparison, the precision drug delivery system might weaken the side effect while strengthening the synthetic lethality at the tumor location as well.

Many drug delivery systems are developed for this purpose. These delivery systems are usually connected with a target, i.e., a membrane protein which is specific or highly expressed on tumor cells. Because of no acknowledged membrane targets still, the development of the drug delivery in osteosarcoma is design restricted, specifically. But non-target drug delivery system might solve this problem. Micro-/nanorobots have been innovatively used to overcome obstacles associated with viscosity forces and Brownian motion and to perform locomotion by using different power (Chen et al., 2018), involving local chemical fuel (Xu et al., 2015; Jurado-Sánchez et al., 2017; Chang et al., 2019; Wu et al., 2019; Mou et al., 2021), external electrical (Wu et al., 2016), optical (Loget and Kuhn, 2011; Dai et al., 2016; Dong et al., 2016; Wu et al., 2016; Xu L. et al., 2017; Tong et al., 2021), ultrasonic (Ding et al., 2012; Wang et al., 2012; Wu et al., 2014; Li et al., 2015; Xu T. et al., 2017; Mu et al., 2021; Zhao et al., 2022), and magnetic (Li T. et al., 2016; Yu et al., 2018; Koleoso et al., 2020; Yang, 2020; Yu et al., 2021; Xu et al., 2022). However, micro-/nanorobots, used as biomedical drug delivery systems, mainly require the elimination of the external fuel (Wang and Pumera, 2015), especially *in vivo* biomedical applications. The robots powered by the chemical fuel, such as hydrogen or enzymolysis response, might be used at the surface of the stomach and intestinal canal. But they cannot be used in the blood or organ tissue (Peyer et al., 2013; Day et al., 2021) because the off-gas of the robots powered by chemical fuel might influence the biofunction of the blood circulation or tissue environment. By comparison, chemical fuel-free robots, which could be driven by a magnetic field, for example, are more biocompatible drug delivery systems (Li et al., 2015; Wu et al., 2018; Ji et al., 2021; Zhang et al., 2021; Liu et al., 2022). Magnetic-driven microrobots could be actively controlled by changing the intensity and direction of the magnetic field. Thus, the permanent magnet or magnetic ion is usually carried. Iron (Fe) (Au et al., 2020; Beladi-Mousavi et al., 2021; Liu et al., 2021), nickel (Ni) (Li et al., 2015; Li et al., 2017a; Li et al., 2017b; Li et al., 2018; Yu et al., 2022), etc. have been used as magnetic propelled materials. Additionally, the fuel-free microrobots could sometimes be driven by double power, such as magnetic–acoustic hybrid power.

Magnetic-driven robots have received tremendous attention in the drug development of tumor treatment. In our previous studies, the magnetic-driven hydrogel microrobot drug delivery system controlled by a directing magnetic field has been indicated (Li et al., 2015; Li J. et al., 2016; Zhang et al., 2021). In this study, a magnetic-driven hydrogel microrobots carried the PRMT5 inhibitor, EPZ015666 (Chan-Penebre et al., 2015), which was synthesized to evaluate a precision synthetic lethality nanodrug for osteosarcoma treatment.

## Materials and Methods

### Cell Lines and Cell Culture

Human osteosarcoma cells used in our study were from ATCC, including 143B and HOS/MNNG. In a previous study, HOS has been demonstrated as MTAP-deleted osteosarcoma and its homologous derivatives, 143B and HOS/MNNG, inherit this feature as well (García-Castellano et al., 2002). All cell lines were stored at Shanghai Bone Tumor Institution (Shanghai, China). Osteosarcoma cells were maintained in DMEM (Wisent Inc., Canada) with 10% FBS (Wisent Inc., Canada) and 1% Penicillin-Streptomycin-Glutamine (Wisent Inc., Canada) in a humidified atmosphere of 5% CO2 and 95% air at 37°C. Cells were dissociated by TrypLE^TM^ Express (Gibco, Thermo Fisher Scientific Inc., United States) and passaged when they are 90–100% confluent. All cell lines were certificated by short tandem repeat (STR) analysis.

### Cell Proliferation Assays

Cells were seeded in 96-well plates at a density of 4000–10000 cells per well and cultured overnight. Then, the drug was added to these 96-well plates and cultured for 72 h or more, and next the supernatant was removed gently. Cell proliferation was detected by the reagent CCK-8 (1:10, with serum-free medium, Dojindo Laboratories, Kumamoto, Japan) according to the manufacturer’s instructions. The absorbance at 450 nm was measured by using a microplate reader SpectraMax M3 (Molecular Devices, Sunnyvale, CA, United States) and Bio-Rad ChemiDoc MP Imaging System. All experiments were performed in triplicate.

### Western Blotting

Total protein was extracted from gathered cells by RIPA protein lysis buffer (Beyotime, Shanghai, China) on cold ice (4°C) in about 30 min. The protein then was collected and centrifuged (12000 rpm) at 4°C for 20 min. The supernatant was collected and the protein concentration of the lysates was measured by the Protein BCA Assay Kit (Bio-Rad, Hercules, CA, United States). For the Western blotting assay, 20 μg of protein mixed with 5 × SDS loading buffer was loaded per lane, separated with 10% SDS-PAGE, and transferred to a polyvinylidene fluoride (PVDF) membrane (MilliporeSigma, Burlington, United States). After blocked with 5% skimmed milk in TBST for 1 h at room temperature, the membrane was incubated at 4°C overnight with primary antibodies (1:1000, PRMT5 #79998, SDMA #13222, GAPDH #5174, Cell Signaling Technology Inc., Danvers, United States), and then, the membrane was washed three times for 10 min with TBS-T solution (TBS, 1 ×; Tween-20, 1:1000) and incubated for 1 h with the corresponding HRP-conjugated secondary antibodies (1:5000, Abgent, San Diego, United States). Chemiluminescent detection was performed using an ECL kit (Pierce Chemical, Rockford, IL, United States) and Bio-Rad ChemiDoc MP Imaging System (Bio-Rad, Hercules, CA, United States). All experiments were performed in triplicate.

### Real-Time PCR

For RT-PCR, EZ-press RNA purification kit (EZBioscience, Roseville, United States) was used to collect the purified total RNA in cultured cells. Then, a color-reverse transcription kit (EZBioscience, Roseville, United States) was applied to remove the genomic DNA (gDNA) and the reverse transcription of RNA to get the cDNA being performed. 2 × color SYBR green qPCR master mix (EZBioscience, Roseville, United States) was used for RT-PCR on QuanStudio six Flex RT-PCR systems (Thermo Fisher Scientific Inc., United States). The protocol was followed and the primers were typed as below:

GAPDH (glyceraldehyde-3-phosphate dehydrogenase):

Human-GAPDH-F GTC​TCC​TCT​GAC​TTC​AAC​AGC​G;

Human-GAPDH-R ACC​ACC​CTG​TTG​CTG​TAG​CCA​A.

MTAP (methylthioadenosine phosphorylase):

Human-MTAP-F GTC​ATA​GTG​ACC​ACA​GCT​TGT​GG;

Human-MTAP-R CCT​CTG​GCA​CAA​GAA​TGA​CTT​CC.

### Synthesis and Characterization of Magnetic-Driven Hydrogel Microrobot Drug Delivery System

Gelatin microparticles (microrobots) were synthesized in a multi-channel fluidic module based on the shear stress of two-phase flow (Figure 3A). 0.5 g of gelatin [Cas (9000–70–8), Aladdin, Shanghai, China] was first dissolved in 10 ml of deionized (DI) water. Then, an additional 2 ml of Fe_3_O_4_ (10–30 nm, 25% in H_2_O, Macklin, Shanghai, China), 1 ml EPZ015666 (10 uM, Cat. No.: HY-12727, MedChemExpress, United States) stained by rhodamine (Macklin, Shanghai, China), and 10 mg 2-hydroxy-4′-(2-hydroxyethoxy)-2-methylpropiophenone (Macklin, Shanghai, China) were added. The mixed solution and dimethyl silicone oil (Macklin, Shanghai, China) were injected into microfluidic chips as sample flow (disperse phase) and sheath flow (continuous phase) using syringe pumps (Harvard Apparatus, United States), respectively. Gelatin microparticles were generated and then irradiated using UV light source. After 10 min of irradiation, the case-hardened gelatin microparticles were obtained, collected by centrifugation, and then washed thrice with DI water to remove oil and any other residue.

### Setup of Magnetic Field

The magnetic field generator consisted of a three-degree-of-freedom Helmholtz coil, multifunction data acquisition unit (DAQ, NI-PCI-6259), and three single-channel output power amplifiers. Based on controlling the current and the voltage of Helmholtz coil from the driving signal amplified by voltage amplifiers, an external rotating uniform magnetic field can be circularly generated in 3D space to manipulate the hydrogel microrobots. The Helmholtz coil was placed on the observation platform of the microscope to achieve real-time observation of swimming hydrogel microrobots. Magnetic dynamics and navigated locomotion of hydrogel microrobots.

The motional performance of the microrobots was investigated under an external magnetic field. A series of control experiments were carried out to elucidate the effect of different drive frequencies and the strength of the magnetic field on the velocity of microrobots. Then, the microrobots were navigated by the control strategy of three-dimensional rotating magnetic field generated by the three-degrees-of-freedom Helmholtz coil. In the x–z plane, the microrobot moved around the short axis (x-axis), applying a magnetic field given by *H* (*t*) = *H*_0_ [cos (*ωt*)e_x_ + sin (*ωt*)] e_z_ (*H*_0_ is the magnitude, *ω* is the angular frequency of the magnetic field, and *t* is the time). Here, e_x_ and e_z_ represent the unit vector along the *x* and *z* axes, respectively (hereafter, e_y_ represents that along the *y* axis). In the y–z plane, the microrobots moved around the long axis (*y*-axis), applying a magnetic field by *H* (*t*) = *H*_0_ [cos (*ωt*)e_y_ + sin (*ωt*)] e_z_. By controlling the input of current, the direction of the rotating magnetic field can be changed to control the direction of the microrobot. Based on the dynamic control scheme above, a predefined trajectory could be walked by a magnetically controlled microrobot.

### *In Vitro* Simulation of Selectively Drug Inhibition of Magnetic Microrobots

We designed an *in vitro* simulated experiment to detect the selective drug inhibition of the magnetic microrobots **(** Figure 5A). We added the microrobots and carried the EPZ015666 into a cell culture dish (35 mm diameter) with a complete medium and then, the microrobots were given a directional magnetic field for 5 min, which was used to simulate the locomotion of microrobots in the blood. When the microrobots moved to one side of the dish, probably 1 min based on the strength of the magnetic field and before the drug released, the liquid close to this side, full of microrobots, was transferred to the 96-well plates cultured with HOS/MNNG cells, which was called “High-dose” group (high dose of microrobots). Meanwhile, the liquid of the other side which lacked microrobots was also transferred to the 96-well plates cultured with HOS/MNNG cells, which was called “Low-dose” group (low dose of microrobots). After treating for 72 h, a CCK-8 assay was given to detect the cell viability of both groups. Also, we used drug-free microrobots and the dissociative drug to repeat the experiment as control.

### Bio-Information and Statistical Analysis

Transcriptomes of patients in The Cancer Genome Atlas (TCGA) and Therapeutically Applicable Research To Generate Effective Treatments (TARGET) databases were downloaded from the Genomic Data Commons (https://portal.gdc.cancer.gov/). The transcriptome of tumor cell lines was downloaded from the Cancer Cell Line Encyclopedia (CCLE) (https://sites.broadinstitute.org/ccle) database. All the data were processed by the same method (R version 4.1.2) and normalized to fragments per kilobase per million (FPKM) using R package edgeR (http://bioconductor.org). The survival rate of patients was analyzed by online tool GEPIA (http://gepia.cancer-pku.cn). Statistical analyses were performed using GraphPad PRISM 8 (GraphPad Software, Inc.) and some visualizations were performed using RStudio 1.4 (https://www.rstudio.com).

## Results and Discussion

### Growth Inhibition of Methylthioadenosine Phosphorylase-Deleted Osteosarcoma by Protein Arginine Methyltransferase 5 Inhibitor

**FIGURE 1:**
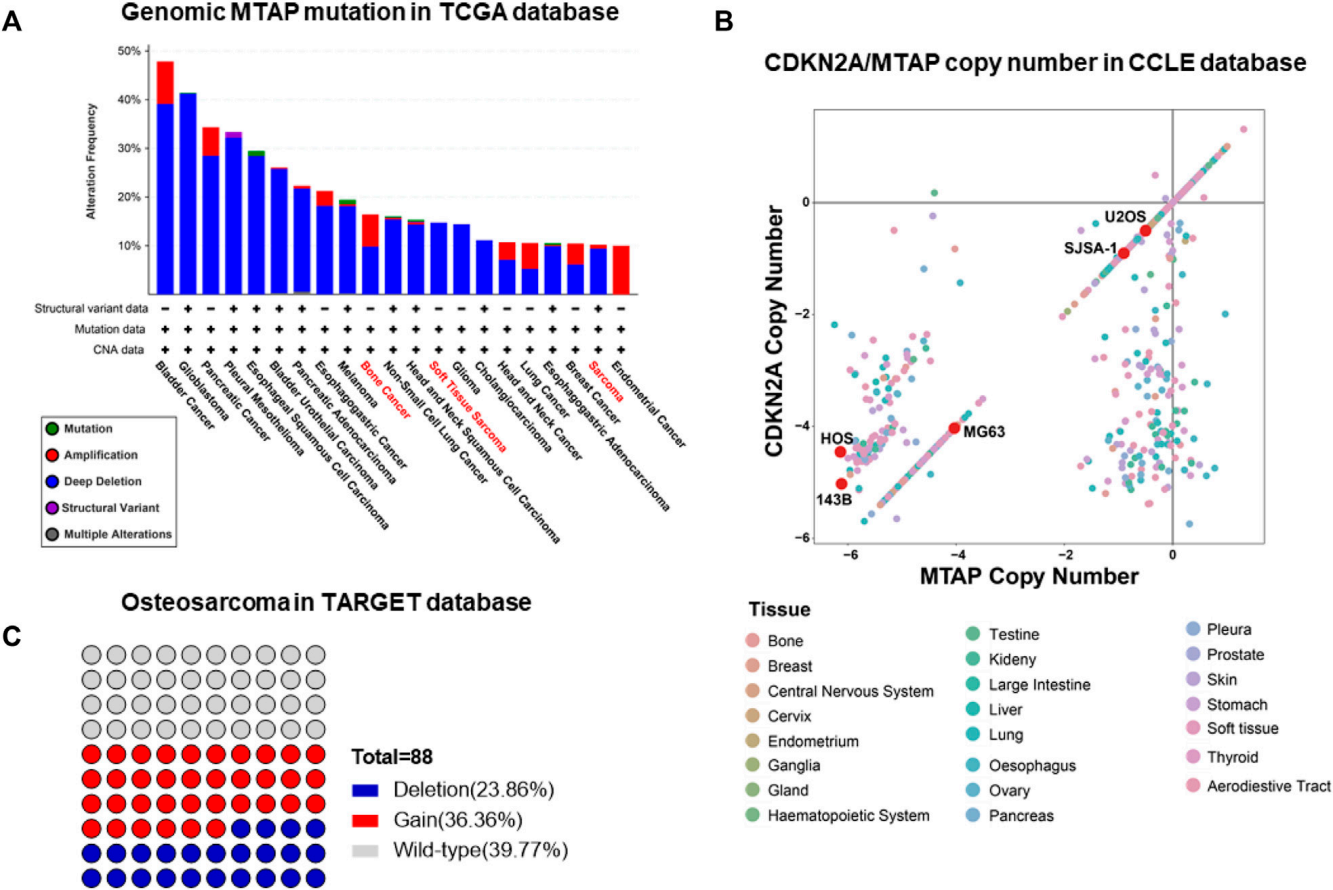
Frequent occurrence of the MTAP deletion in osteosarcoma. **(A)** Genomic mutation of MTAP in pan-cancer in the TCGA database. **(B)** Copy number of CDKN2A and MTAP in pan-cancer cell lines in the CCLE database. **(C)** Genomic mutation of MTAP in osteosarcoma in the TARGET database.

**FIGURE 2:**
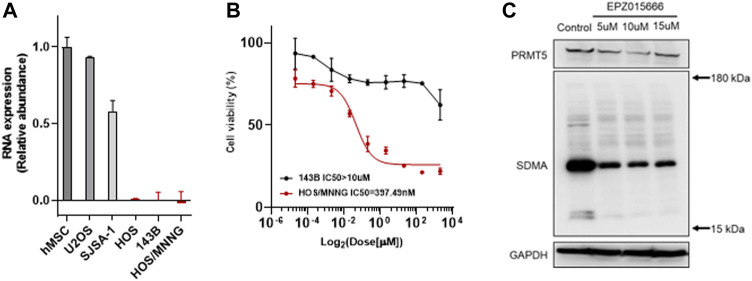
EPZ015666 inhibits the growth of MTAP-deleted osteosarcoma. **(A)** Relative abundance of MTAP RNA expression in hMSCs and common osteosarcoma cell lines (U2OS, SJSA-1, HOS, 143B and HOS/MNNG) *in vitro*, respectively. **(B)** Cell viability of MTAP-deleted osteosarcoma (black and red line represents 143B and HOS/MNNG, respectively) after using a concentration gradient of EZP015666 *in vitro*. **(C)** SDMA modification reduction by treated EPZ015666 (143B) *in vitro*. hMSC, human mesenchymal stem cell; IC50, half maximal inhibitory concentration; SDMA, symmetric di-methyl-arginine.

The enhancement or deficiency of gene function leads to cellular changes in various aspects and results in tumor generation or malignancy, respectively. The genomic mutation on the one hand, gives the tumor cell advantages in competitive growth, on the other hand, foreshadows hide vulnerabilities in tumor cells. Following this, some unexpected gene relationships have been found by CRISPR-cas9 screening and the synthetic lethality gene has been demonstrated as a vulnerability to a specific mutation in pan-cancer genomic analyses. Genomic mutation of MTAP deletion occurred commonly in pan-cancer in the TCGA database and there was nearly 10% MTAP deletion in sarcoma (Figure 1A). Methionine metabolism in tumor cells is disordered by MTAP deletion at chromosome 9p21.3. To further confirm the frequency of MTAP deletion in osteosarcoma, the pan-cancer cell lines in the CCLE database and the tumor tissue in the TARGET database were detected. Then, by comparing the two main genes located at chromosome 9p21.3, CDKN2A and MTAP, and two homologous cell lines of all osteosarcoma cell lines, 143B and HOS, the lowest copy number of CDKN2A and MTAP were shown in Figure 1B. In the TARGET database, 23.86% of osteosarcoma revealed genomic MTAP deletion, including the loss of heterozygosity (LoH) and homologous deletion (Figure 1C). The results have demonstrated that MTAP deletion happens frequently in osteosarcoma. As a result, the intracellular 5′-methylthioadenosine (MTA) cannot be metabolized because of the loss of MTAP function which leads to the tumor being dependent on PRMT5. Then, MTAP RNA expression in the hMSC (human mesenchymal stem cell) and common osteosarcoma cell lines (U2OS, SJSA-1, HOS, 143B, and HOS/MNNG) were further detected. The mRNA expression of U2OS and SJSA-1 was like hMSC, but HOS and its derivatives, 143B and HOS/MNNG, were loss of the mRNA expression of MTAP. Thus, the MTAP function was lost as indicated by the results (Figure 2A) and they, called MTAP-deleted osteosarcoma, might be sensitive to the PRMT5 inhibitor. PRMT5 inhibitor, EPZ01566, reduces the level of SDMA (symmetric di-methyl-arginine) modification and induces DNA damage to MTAP-deleted tumor cells and blocks tumor growth (Kalev et al., 2021). Therefore, the effect of PRMT5 inhibition on tumor growth was next detected. The cell viability of 143B and HOS/MNNG were influenced by EPZ015666, a PRMT5 inhibitor (Figure 2B). Considering whether it was related to the inhibition of the function of PRTM5 enzyme, we detected SDMA, the modification site of PRMT5, which was reduced by PRMT5 inhibition for 72 h (Figure 2C). To conclude, our results have demonstrated that PRMT5 inhibitor, EPZ015666, could block the growth of MTAP-deleted osteosarcoma and inhibits the level of SDMA modification *in vitro*. The growth inhibition of EPZ01566 to MTAP-deleted osteosarcomas is different on 143B and HOS/MNNG, while HOS/MNNG seems to be more sensitive to PRMT5 inhibition *in vitro*.

### Preparation and Drug Efficiency of Magnetic-Driven Hydrogel Microrobot Drug Delivery System *In Vitro*

**FIGURE 3:**
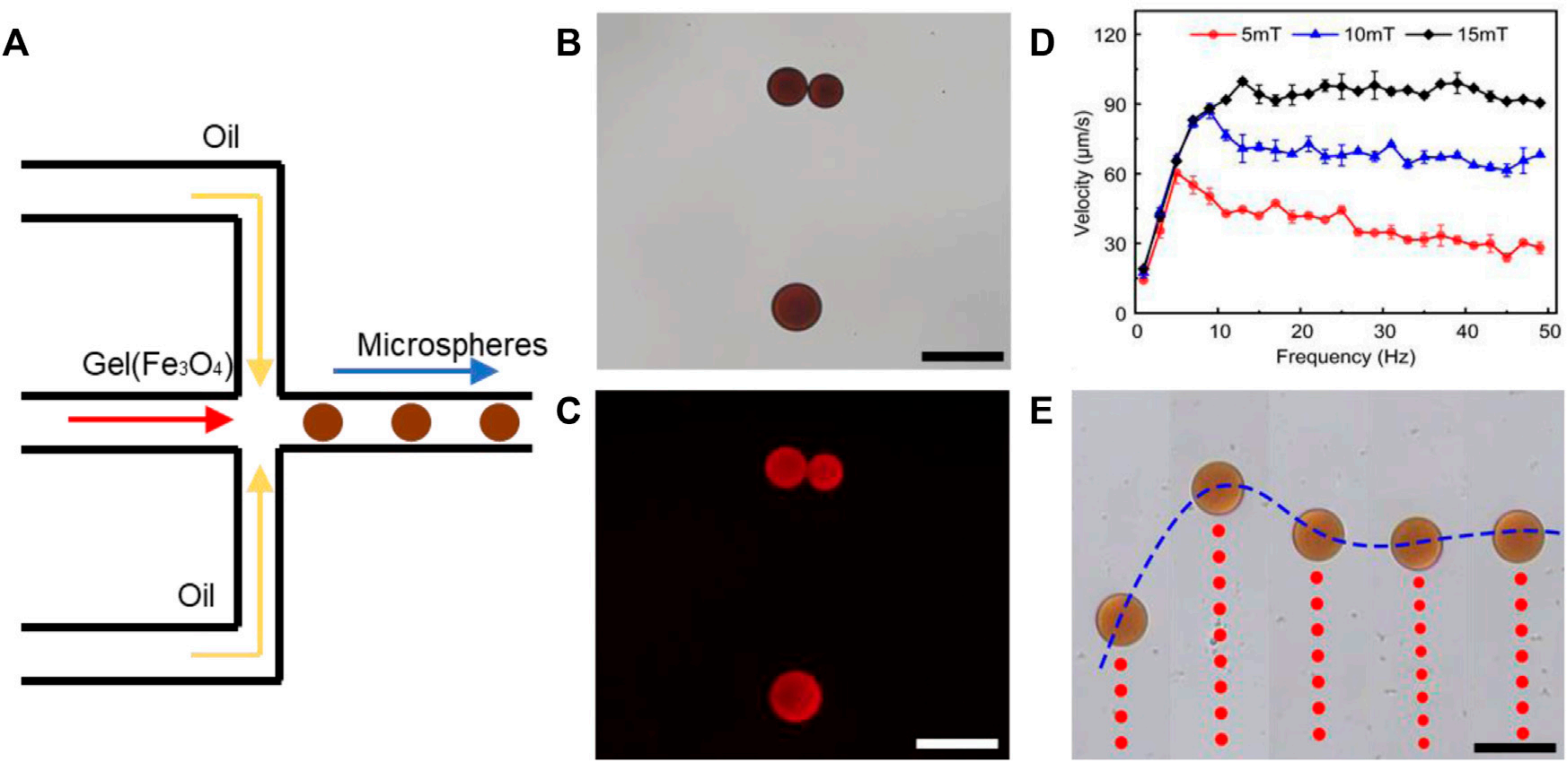
Synthesis and characterization of magnetic-driven hydrogel microrobots. **(A)** Schematic of the microrobot synthesis. **(B)** Microrobot characteristics under bright field. **(C)** Microrobot characteristics under dark field and the EPZ015666 stained by rhodamine was detected as red fluorescence. **(D,E)** By enhancing the intensity and frequency of the magnetic field, the velocity of microrobots was growing faster. Scale bar of **B**,**C**, and **E** = 50 μm.

**FIGURE 4:**
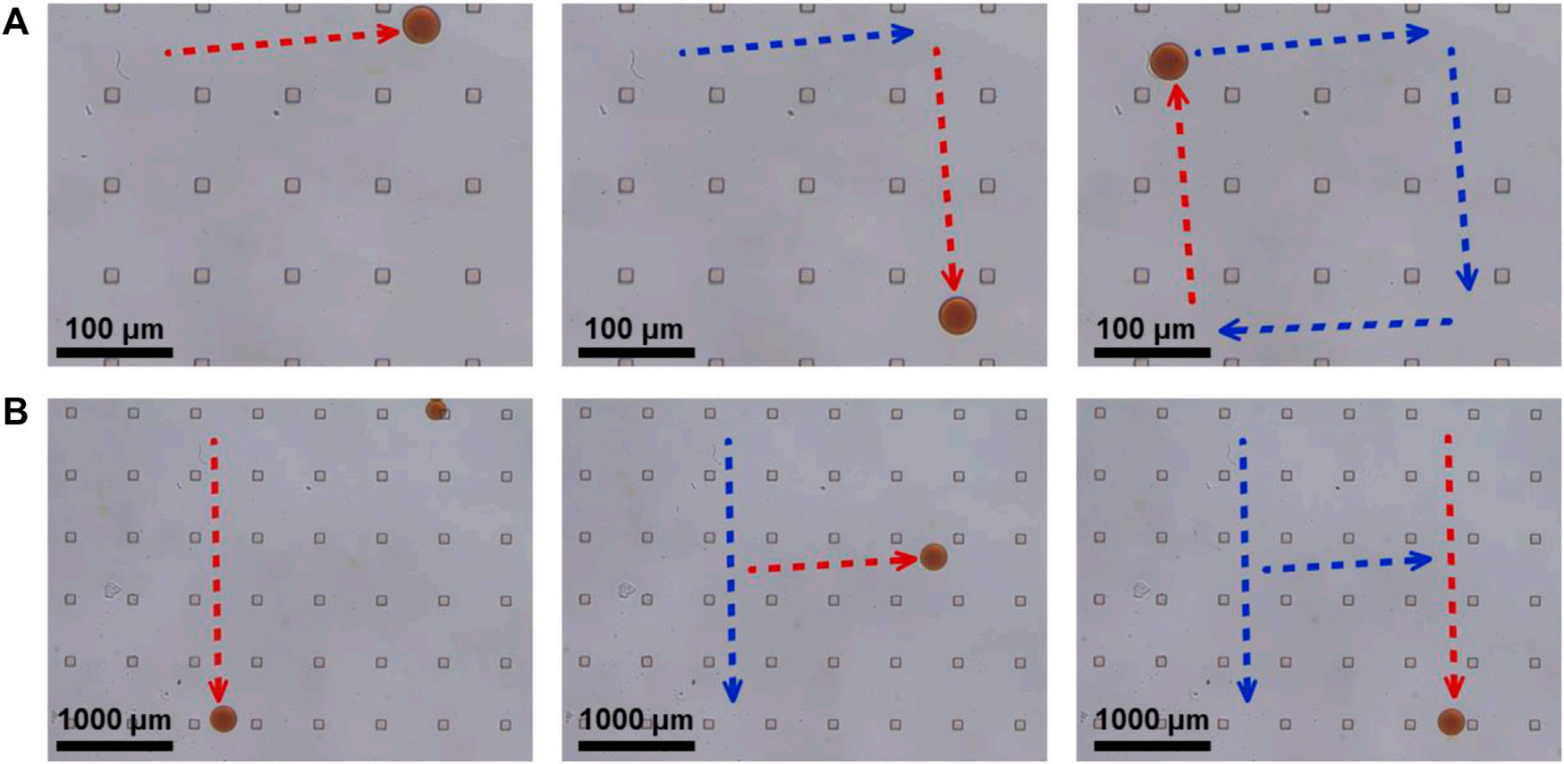
Predetermined locomotion of magnetic-driven hydrogel microrobots. **(A)** The microrobot was driven by the predetermined direction of a square. **(B)** The microrobot was driven by the predetermined direction of letter H.

**FIGURE 5:**
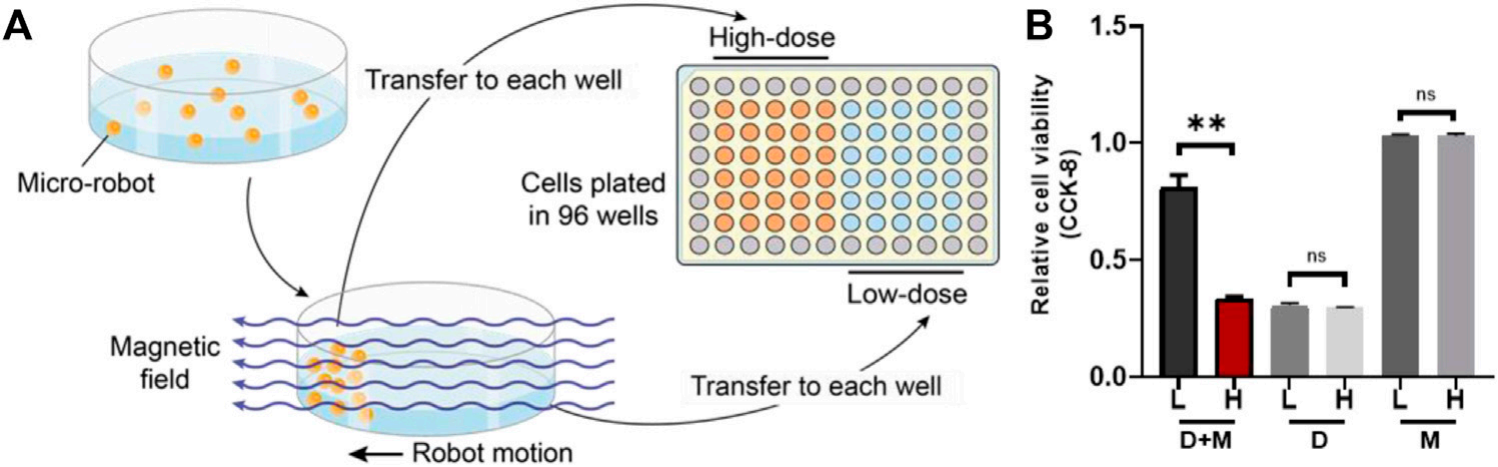
The simulation of selectively drug inhibition of microrobots *in vitro*. **(A)** The sketch map of simulation. Most of the microrobots were driven by the magnetic field to one side of the culture dish and then the liquid was added to both sides (low-dose and high dose sides of microrobots) in the 96-well plate culturing HOS/MNNG tumor cells. **(B)** A lower cell viability was detected in the high dose of microrobots (**: *p* < 0.01). L, Low-dose; H, High-dose; D + M, Microrobots with EPZ015666; D, Dissociative EPZ015666; M, Drug-free microrobots.

Theoretically, synthetic lethality drugs inhibit the growth of typical tumor cells without affecting normal cells. However, the synthetic lethality drug can also combine the target with normal cells (Wang et al., 2018). Although it slightly affects normal cells, the drugs arriving at the tumor location decrease and this gentle inhibition of tumor cells might fail. Also, side effects might appear with increasing the dose of such drugs. However, a drug delivery system might be useful for synthetic lethality drugs to reduce the side effects. Non-target drug delivery system based on magnetic-driven hydrogel microrobots could not only solve this problem but also avoid the obsession that no specific membrane protein is acknowledged to be the target of osteosarcoma. Microrobots, now being used for a multitude of applications, such as drug delivery, surgery, and diagnosis, can easily overcome the blood flow and approach the tumor loci actively, with the power of chemical or physical engine (Wu et al., 2021). Magnetic-driven microrobots are generated by moving charges and magnetic materials, and thus capable of moving to the scheduled destination by the navigation of magnetic fields (Mujtaba et al., 2021; Zhang et al., 2021; Zhou et al., 2021). In this study, a magnetic-driven hydrogel microrobot for synthetic lethality drug delivery system has been innovated, while the morphological characteristics of microrobots by electron microscopy have been detected as well. The diameter of magnetic-driven hydrogel microrobots was about 30 μm. The microrobots under bright field showed dark red (Figure 3B), which carried EPZ015666 stained by rhodamine and Fe_3_O_4_, and under dark field showed bright red fluorescence, counting about 200 microrobots per microliter (Figure 3C). This result revealed that the EPZ015666 was successfully loaded by microrobots. Then, the motion of the microrobots under magnetic field was detected. It has been observed that the velocity of microrobots was growing faster by enhancing the intensity and frequency of the magnetic field, and the highest velocity is 100 μm/s (Figures 3D,E). For predetermined locomotion experiments, the microrobots were driven by the predetermined direction of the magnetic field and tracked a square (Figure 4A and Supplementary Video S1) or letter H (Figure 4B and Supplementary Video S2). Our results have demonstrated that the microrobots carry the EPZ01566, stained by the rhodamine, and the movement of microrobots could be controlled by changing the magnetic field. Our drug delivery system could sustainedly release the drug into the complete medium for 15–20 min completely. The sustained-release microrobots are supposed to reduce the side-effect to normal cells and enhance the drug lethality in tumor cells (Chen et al., 2021). The selected inhibition of the magnetic-driven hydrogel microrobot drug delivery system was simulated *in vitro* (Figure 5A). Most of the microrobots were driven by the magnetic field to one side of the culture dish, a diameter equal to 35 mm for nearly 5 min. Then, by adding the liquid of this side (High-dose: high dose of microrobots) and the opposite side (Low-dose: low-dose of microrobots) in the 96-well plate culturing HOS/MNNG tumor cells, lower cell viability was detected in the high dose of microrobots (Figure 5B). In a simulated *in vitro* experiment, it has been indicated that the magnetic-driven microrobots could carry drugs and inhibit tumor growth. As a result, our magnetic-driven hydrogel microrobot drug delivery system can be used for the non-targeted therapy of MTAP-deleted osteosarcoma. But there is still a big gap between research and development and the clinical implication of treating osteosarcoma. The patients who unluckily suffered the osteosarcoma usually have a large tumor load. The locomotion of the microrobots in the solid sarcoma might be more difficult than in the blood cancer due to the difference in the medium. The microrobots overcoming the resistance to the blood flow and driven to the tumor site could be achievable, but the locomotion going further into the deeper site of the primary tumor seems to need further study. Our study will further verify whether the magnetic microrobots carried out by typical synthetic lethal drugs could inhibit the growth of osteosarcoma *in vivo* under the guidance of genomic features.

## Conclusion

In this study, a new magnetic-driven hydrogel microrobot for the delivery of one PRMT5 inhibitor, EPZ01566, has been demonstrated. The microrobots, as a non-target drug delivery system, selectively enhance the drug efficiency of synthetic lethality drugs, was attempted. Our drug delivery system aimed to carry the typical synthetic lethal drug to enhance the inhibition of the growth of the osteosarcoma in primary tumors under the guidance of genomic features. Our microrobots, which carry EPZ015666 stained by rhodamine, could be actively controlled by changing the external magnetic field. These microrobots could selectively inhibit the tumor growth of the MTAP-deleted osteosarcoma in a simulation *in vitro*. Since MTAP deletion happens frequently in osteosarcoma and its synthetic lethality pair PRMT5 inhibitor might target normal cells *in vivo*, our microrobots might become a new delivery approach to deliver the synthetic lethal drug directly to the tumor site to enhance the drug efficiency and reduce the side effects.

## Data Availability

The raw data supporting the conclusions of this article will be made available by the authors, without undue reservation.
